# Stable responses to danicopan as add-on to ravulizumab in two patients with paroxysmal nocturnal hemoglobinuria

**DOI:** 10.1007/s00277-024-05858-x

**Published:** 2024-06-26

**Authors:** Wolfgang Füreder, Peter Valent

**Affiliations:** 1 Department of Internal Medicine I, Division of Hematology & Hemostaseology, Medical University of Vienna, Vienna, Austria; 2 Ludwig Boltzmann Institute for Hematology and Oncology, Medical University of Vienna, Vienna, Austria

**Keywords:** PNH, MDS, Ferritin, Hemolysis

## To the editor

Paroxysmal nocturnal hemoglobinuria (PNH) is an acquired stem cell disease defined by the absence of glycosylphosphatidylinositol (GPI)-linked surface proteins, such as CD55 and CD59, on various hematopoietic cells. Absence of CD55 and CD59 on the surface of PNH erythrocytes leaves them susceptible to hemolysis. Typical findings in PNH patients include anemia, fatigue, and thrombophilia. Currently, blockage of the complement cascade using the C5 inhibitors ravulizumab or eculizumab is the standard of care. However, in a subset of PNH patients treated with C5 inhibitors, extravascular hemolysis develops. This may lead to transfusion-dependent anemia and chronic fatigue [1]. In most patients with extravascular hemolysis (EVH), anemia is accompanied by low haptoglobin levels and increased reticulocyte counts. The optimal management of such patients remains a clinical challenge.

Danicopan is a novel “complement-targeting” drug that inhibits factor-D in the alternative pathway of the complement system. This drug has shown promising results in recent clinical trials [2, 3]. However, so far, no real world data with danicopan have been collected in PNH patients.

We treated two patients in a named patient program with danicopan as an add-on to ravulizumab. Both patients remained anemic during single agent C5 inhibition, had high reticulocyte counts, and suffered from considerable fatigue before the start of danicopan (Table 1 and Table 2).

**Table 1:** Patient characteristics - patient 1

	BL*	w 1	w 2	w 3	w5	w7	w10	w12	w15	w17	w19
Hemoglobin, g/dL	7.8	11.1	11.5	11.5	11.5	11.3	10.6	10.8	10.7	10.9	11.2
FACIT fatigue score	27	nd	nd	nd	47	46	34	36	41	42	43
Reticulocytes, 10^9^/L	306.4	144.2	42.2	35.0	53.4	67.2	119.9	117.4	122.1	110.9	119.0
White blood count, 10^9^/L	2.38	1.64	2.08	2.35	2.10	2.42	2.02	2.61	2.04	2.26	2.24
Absolute neutrophil count, 10^9^/L	1.1	0.6	0.9	1.2	1.0	1.0	0.8	1.2	0.8	1.0	1.0
Platelet count, 10^9^/L	95	68	71	84	81	85	82	75	70	81	76
Erythrocyte-clone size, %	38	65	71	75	83	88	96	98	100	100	100
Granulocyte-clone size, %	100	100	100	100	100	100	100	100	100	100	100
Ferritin, µg/L	1727	nd	823.2	nd	574.7	697.4	670.1	551.2	538.9	522.0	400.3
Transferrin saturation, %	46.4	nd	23.6	nd	28.1	35.8	28.2	26.5	27.5	25.6	25.3
Bilirubin, mg/dL	0.79	0.59	0.47	0.48	0.42	0.41	0.52	0.4	0.42	0.46	0.44
LDH, U/L	368	266	283	263	272	302	330	307	325	337	341

BL, baseline; w, week; LDH, lactate-dehydrogenase; nd, not done. * Last FACIT fatigue score prior to danicopan was recorded 3 years before start of danicopan. All other data for BL were collected one week prior to danicopan

Patient 1 was a 70-year-old female patient diagnosed in 2004 with PNH and an associated myelodysplastic syndrome (MDS) subtype “refractory anemia” according to the French-American-British (FAB) classification and “MDS with low blasts” (MDS-LB) according to the WHO classification 2022. In 2004, the international prognostic scoring system (IPSS) revealed an intermediate 1 risk. Cytogenetics showed a normal female karyotype: 46,XX. Complement inhibition was initiated in 2013 using eculizumab and was changed to ravulizumab in 2019. Other medications included deferasirox and deferoxamine for iron overload, pregabalin and trazodone-hydrochloride for anxiety and depression, and folic acid. Since her endogenous serum erythropoietin level was adequate for hematocrit, no erythropoietin therapy was given. During the previous 12 months prior to the initiation of danicopan, she had received 16 packed red cell transfusions. After informed consent was obtained, the patient received 150 mg danicopan three times a day (tid) orally as add-on to ravulizumab 3300 mg, every 8 weeks.

Patient 2 was a 23-year-old female diagnosed with PNH in 2017. She also suffered from a Budd Chiari syndrome in 2017 that was treated with spironolactone, carvedilol, and anticoagulation with acenocoumarin. Therapy with eculizumab was initiated in 2018 and was switched to ravulizumab in 2019. The patient also received iron supplementation and folic acid. She had received 2 red cell transfusions in the last 12 months before danicopan was started. After informed consent was given, the patient received 150 mg danicopan tid as add-on to ravulizumab 3300 mg every 8 weeks.

**Fig. 1:**
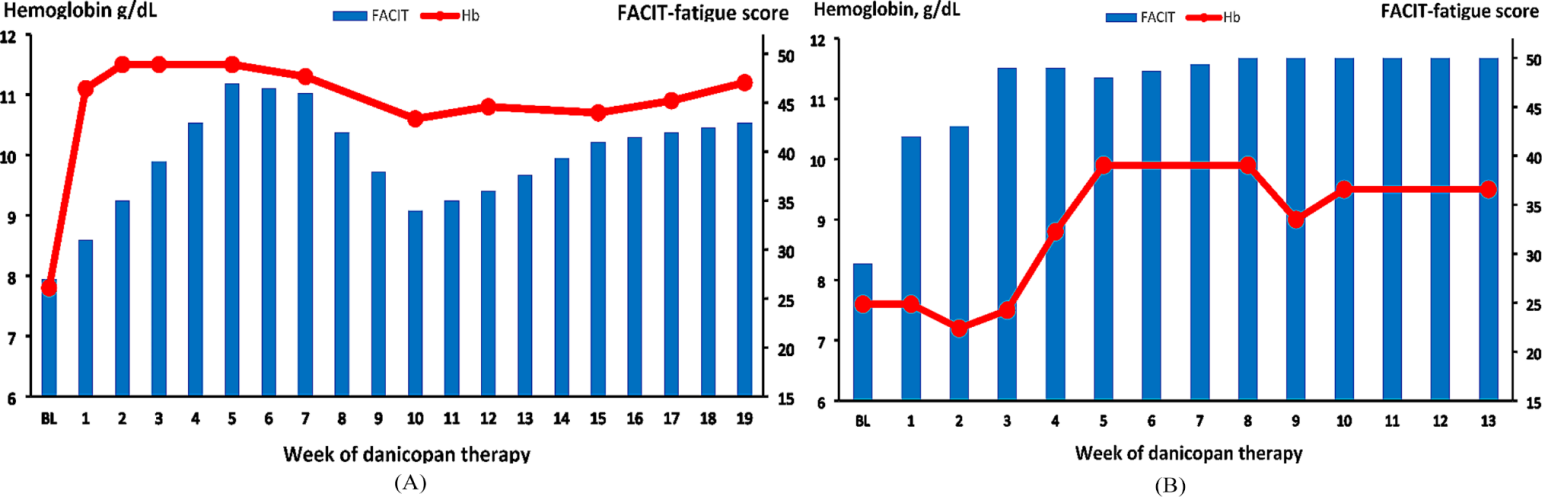
(**a**) Hemoglobin level (red line) and FACIT-fatigue score (blue bars) in patient (1) After the start of danicopan, hemoglobin increased rapidly in this patient and the FACIT-fatigue score rose by 20 points compared to baseline. Please note that FACIT-fatigue score baseline was 3 years prior to initiation of danicopan, whereas hemoglobin baseline was one week before start of therapy. Hb, hemoglobin; FACIT, FACIT-fatigue score; BL, baseline. (**b**) Hemoglobin level (red line) and FACIT fatigue score (blue bars) in patient (2) While the FACIT fatigue score increased rapidly in this patient, the increase of hemoglobin was more delayed, probably due to iron deficiency. Once iron deficiency was corrected, hemoglobin increased also substantially as compared to baseline. Timepoint of baseline was three weeks prior to danicopan start in this patient for hemoglobin and day 0 for FACIT fatigue score. Hb, hemoglobin; FACIT, FACIT-fatigue score; BL, baseline

Following the start of danicopan therapy, the hemoglobin level of patient 1 increased rapidly and reticulocyte counts decreased within a few weeks (Fig. 1A; Table 1). Her FACIT fatigue score improved from 27 before danicopan to a peak level of 47 during danicopan (Fig. 1A). The PNH erythrocyte clone increased in size from 38% pre-danicopan gradually to 100% within 15 weeks (Table 1). Blood chemistry remained normal apart from an ongoing minor LDH elevation (< 1,5xULN). At week 8 of danicopan therapy, the patient suffered from a respiratory infection and in week 9 she received a meningococcal vaccine booster. Shortly thereafter, her hemoglobin decreased slightly, her reticulocyte counts increased, and her FACIT score dropped by 12 points (Fig. 1A; Table 1). LDH remained < 1.5xULN at that time, and during the following weeks, hemoglobin levels and reticulocyte counts stabilized (Table 1; Fig. 1A).

**Table 2:** Patient characteristics - patient 2

	BL*	w1	w2	w3	w4	w5	w8	w9	w10	w13
Hemoglobin, g/dL	7.6	7.6	7.2	7.5	8.8	9.9	9.9	9.0	9.5	9.5
FACIT-fatigue score	29	42	43	49	49	48	50	50	50	50
Reticulocytes, 10^9^/L	180.0	161.3	156.7	169.7	251.8	194.3	205.1	228.2	218.1	178.5
White blood count, 10^9^/L	2.74	4.48	3.21	3.28	3.28	2.99	3.27	4.11	3.26	2.79
Absolute neutrophil count, 10^9^/L	1.5	nd	2.0	1.8	1.9	1.8	nd	2.3	2.0	nd
Platelet count, 10^9^/L	78	80	67	70	58	60	72	74	70	63
Erythrocyte-clone size, %	74	64	78	83	89	87	79	89	86	86
Granulocyte-clone size, %	74	80	83	83	82	84	81	82	83	82
Ferritin, µg/L	15.7	22.8	< 15	41	246.8	283.7	225.9	81.4	52.5	58.0
Transferrin saturation, %	11.2	5	11	8.9	17.4	25.3	88.8	28.1	14.4	25.5
Bilirubin, mg/dL	4.05	1.91	1.46	1.81	1.77	1.31	6.88	1.24	1.25	1.75
LDH, U/L	211	150	110	133	122	121	330	144	153	126

BL, baseline; w, week; LDH, lactate-dehydrogenase; nd, not done. *Data for BL were collected three weeks prior to danicopan with the exception of the FACIT fatigue score that was evaluated one week before danicopan. Erythrocyte-and granulocyte- clone sizes were analyzed immediately before danicopan at day 0

Interestingly, patient 1 who received iron chelation therapy because of transfusion-induced iron overload, showed a steep drop in ferritin after the start of danicopan. Despite treatment with deferasirox, her ferritin level at baseline was 1727 µg/L. Within two weeks of danicopan treatment, ferritin decreased to 823,2 µg/L and three weeks later to 574,7 µg/L (Table 1). This might be explained by an increased iron incorporation into red cells that escaped degradation due to danicopan therapy.

Patient 2 suffered from concomitant iron deficiency, probably due to heavy menstrual bleeding combined with thrombocytopenia and was on a therapy with oral anticoagulation for Budd-Chiari syndrome. Recently published data support the conclusion that discontinuation of anticoagulation may be considered in such patients provided that imaging studies disclose negative results and hemolytic parameters can be kept under control [4]. In our patient, MRT-scan of the liver during follow up revealed cirrhosis and liver veins were not detectable. Therefore, anticoagulation was continued. Despite oral iron supplementation, ferritin remained low after initiation of danicopan. Subsequently, the patient received intravenous iron therapy. During danicopan, haptoglobin normalized and bilirubin decreased substantially. In addition, the patients’ FACIT fatigue score increased by more than 10 points (Fig. 1B). Probably due to iron deficiency, the increase of hemoglobin after initiation of danicopan was more delayed in this patient. In fact, a substantial increase in hemoglobin was only seen after the start of intravenous iron supplementation in week 4 (Fig. 1B).

Like in patient 1, the hemoglobin level in patient 2 dropped slightly around week 9 after reaching a peak at week 8, and reticulocyte counts increased (Fig. 1B; Table 2). Moreover, at this timepoint the bilirubin level spiked, and haptoglobin that had normalized in the first few weeks, dropped again below the detection limit. In addition, LDH increased. Subsequently, haptoglobin normalized, bilirubin and LDH dropped again, and the hemoglobin level stabilized (Fig. 1B; Table 2).

Breakthrough-hemolysis (BTH) might be a condition more pronounced in patients treated with proximal complement inhibition alone [5, 6]. With dual complement inhibition, the risk to develop a relevant BTH may be lower [3]. Both of our patients developed hemolysis (starting at week 10 in patient 1; and at week 8 in patient 2). However, these hemolytic episodes were mild and transient, and did not meet established criteria for BTH [7]. In particular, no substantial increase in LDH was observed. One reason for the hemolytic episode in patient 1 may be the combination of two complement-amplifying events: a respiratory infection and a vaccination booster. Whether the hemolytic events would have caused a more severe BTH if the patients had only received C5 inhibition as monotherapy remains unknown.

An alternative explanation for the reappearance of some degree of hemolysis might be a compliance issue with danicopan intake. However, the patients strongly insisted that they had taken medication as prescribed. As a result, we believe that adherence to therapy with danicopan was appropriate in our patients. In fact, the FACIT fatigue scores showed a dramatic improvement of fatigue in both of our patients. It is worth noting in this regard that patients who experience an immediate benefit of proximal complement inhibition as add-on to C5 inhibition are likely to adhere to such therapy. Unfortunately, due to the unavailability of danicopan serum level measurement at our institution, we were unable to clarify this issue.

Another possibility for the transient decrease in hemoglobin in both patients and fatigue in patient 1, could be a diminished effectiveness of danicopan therapy over time or some kind of ‘escape mechanism’ by which the complement system circumvents blockage of factor D by danicopan. It is also noteworthy, that our observations regarding a slight decrease in hemoglobin and increase in reticulocyte counts several weeks after the start of danicopan are in line with published reports [3]. Finally, it should be mentioned that in a trial, the dose of danicopan tid was increased from 150 mg tid to 200 mg tid in order to achieve investigator desired clinical responses [3]. We also considered a possible increase of danicopan to 200 mg tid. However, since hemoglobin levels did not further decrease and fatigue did not worsen, we finally decided not to modify the doses of danicopan.

Whether dual inhibition poses an increased risk for severe infections remains currently unknown. In most trials assessing proximal complement inhibitors, these drugs were added to C5 inhibitors, and only later, proximal complement blockers were tested as monotherapy. In all these trials, no increased risk for infections were reported [3, 8, 9]. Whether in the long-term, infections are more common in patients treated with dual complement inhibition (or even in monotherapy with proximal inhibitors) remains to be determined. It also remains unknown whether an additional MDS may predispose even more to severe infections, due to neutropenia and altered MDS neutrophil´s function.

Together, in our patients, hemoglobin and FACIT-fatigue scores improved substantially after initiation of danicopan therapy. Both patients remained transfusion-free, and no treatment-related side effects were reported.

## Data Availability

No datasets were generated or analysed during the current study.
